# Angiogenesis and Endometriosis

**DOI:** 10.1155/2013/859619

**Published:** 2013-05-26

**Authors:** Ana Luiza L. Rocha, Fernando M. Reis, Robert N. Taylor

**Affiliations:** ^1^Department of Obstetrics and Gynecology, Division of Human Reproduction, Federal University of Minas Gerais, 310130-100 Belo Horizonte, MG, Brazil; ^2^Department of Obstetrics and Gynecology, Wake Forest School of Medicine, Winston-Salem, NC 27157-1066, USA

## Abstract

A comprehensive review was performed to survey the role of angiogenesis in the pathogenesis of endometriosis. This is a multifactorial disease in which the development and maintenance of endometriotic implants depend on their invasive capacity and angiogenic potential. The peritoneal fluid of patients with endometriosis is a complex suspension carrying inflammatory cytokines, growth factors, steroid hormones, proangiogenic factors, macrophages, and endometrial and red blood cells. These cells and their signaling products concur to promote the spreading of new blood vessels at the endometriotic lesions and surroundings, which contributes to the endometriotic implant survival. Experimental studies of several antiangiogenic agents demonstrated the regression of endometriotic lesions by reducing their blood supply. Further studies are necessary before these novel agents can be introduced into clinical practice, in particular the establishment of the safety of anti-angiogenic medications in women who are seeking to become pregnant.

## 1. Introduction

Endometriosis is a benign sex hormone-dependent gynecological disease, characterized by the presence and growth of endometrial tissue outside the uterus; it affects 10% of women of reproductive age and is associated with infertility and pain [1, 2]. The symptoms can impact on general physical, mental, and social well-being [3]. Despite many investigations about endometriosis, the pathogenesis of the disease remains unclear [3]. The disease derives from retrograde menstruation of endometrial cells which implant on peritoneal surfaces and induce an inflammatory response. The success of the ectopic implants depends on other pathological processes such as neoangiogenesis, fibrosis, adhesion formation, avoidance of apoptosis, immune dysfunction, and neuronal infiltration [1, 2, 4–7].

During normal reproduction, cyclic angiogenesis is orchestrated by the endocrine system, providing physiological signals for follicular maturation, corpus luteum function, endometrial growth, and remodeling [8]. Endometriosis is a multifactorial disease in which angiogenesis also plays an important role [9–13]. The angiogenic potential of both the endometrium and the peritoneal environment influences lesion establishment [9–12]. Indeed, endometriotic lesions require an adequate blood supply to survive in their ectopic sites. 

The goals of endometriosis treatment alternate between alleviation of pelvic pain and successful achievement of pregnancy in infertile patients. Antiangiogenic drugs hold a promise for both indications and present a distinct perspective in endometriosis treatment. 

The aim of this paper is to review the literature evidence of the important role of angiogenesis in the pathogenesis of endometriosis and to establish the rationale for anti-angiogenic agents as a new therapeutic option in the treatment of endometriosis patients.

## 2. Methods

### 2.1. Search Strategy

A literature search was performed to survey the role of angiogenesis in the pathogenesis of endometriosis. Articles were identified through the following electronic databases: MEDLINE (until January 2013) and the Cochrane Central Register of Controlled Trials (The Cochrane Library until January 2013). A combination of Medical Subject Headings (MeSH) and text words was used to generate the list of citations: (endometriosis OR “endometriotic lesions”) AND (angiogenesis OR “angiogenic factors” OR vasculogenesis OR “antiangiogenic drugs”). All pertinent articles were examined and their reference lists were reviewed in order to identify other studies for potential inclusion in this review. No institutional review board approval was required because only published data were analyzed.

### 2.2. Selection Criteria

Randomized controlled trials (RCTs), patient preference trials, observational studies, case reports, and proceedings of scientific meetings were included in this review, whereas abstracts were excluded. Only publications in English were considered in our selection. The abstracts of studies identified in the search were reviewed to exclude irrelevant or repeat citations. The reviewers were not blinded to the names of investigators or sources of publication.

## 3. Results

### 3.1. Angiogenesis in Endometrium and in Endometriotic Implants

Endometriotic lesions are typically characterized by a dense vascularization that occurs through angiogenesis process [1, 9, 14]. In normal eutopic (intrauterine) endometrium, it has been suggested that vessel elongation, rather than branch point sprouting, is the primary mechanism for rapid vessel growth during the proliferative phase [15], but the precise mechanism in endometriosis lesions has not been evaluated to date. Recruitment of new capillaries from existing, adjacent peritoneal microvessels was postulated [10]; however, the derivation of new blood vessels from circulating endothelial progenitor cells (EPCs), the so-called “vasculogenesis,” also appears to be important in the pathogenesis of endometriosis [14]. The endometrium is a dynamic tissue exhibiting populations of clonogenic epithelial and stromal stem cells [16–18] that require active cyclic angiogenesis. Bone-marrow-derived EPCs can be detected in developing endometriotic lesions [19] and those lesions show increased expression of factors and chemokines that participate in EPC recruitment, such as hypoxia-inducible-factor- (HIF-) 1*α* and stromal-cell-derived-factor- (SDF-) 1 [14, 20]. Moreover, the presence of hypoxia, endothelial injury, and inflammation and the expression of ER-*α* contribute to the mobilization and recruitment of EPCs from the bone marrow into endometriotic lesions [14, 21–27].

Endometriotic lesions can produce cytokines and growth factors that regulate their proliferation and vascularization. Interleukin- (IL-) 1*β*, the dominant IL-1 secreted by activated peritoneal macrophages, plays an important role in the neovascularization of endometriotic lesions [28, 29]. Cultured human endometrial stromal cells (HESC) from women with endometriosis secrete IL-6 and IL-8 robustly [30]. IL-6 is a potent multifunctional protein, which promotes endometrial cell proliferation [31] and angiogenesis [32]; its secretion is elevated in ectopic endometrial tissue and its concentrations are high in peritoneal fluid of patients with endometriosis [33]. IL-8 is a proinflammatory cytokine that induces chemotaxis of neutrophils and has a potent stimulatory effect on angiogenesis [34, 35].

Activin A is a growth factor member of the transforming growth factor *β* superfamily with effects on inflammation and angiogenesis [36–38]. The human endometrium is both a source and a target of activin A, which is able to modulate the expression and secretion of IL-8 and vascular endothelial growth factor (VEGF), from human endometrial stromal cells [39]. VEGF is among the most potent and specific angiogenic factors. Its effects include endothelial cell proliferation, migration, organization into tubules, and enhanced permeability, all of which participate in the angiogenic cascade [40]. Endometrial VEGF expression is enhanced by estradiol and its concentrations are correlated with neovascularization and increased vascular permeability during late proliferative phase [41]. Cyclic changes in VEGF expression throughout menstrual cycle are observed with maximal expression during the secretory phase and menstruation [9, 41, 42]. VEGF was observed in the epithelium and in stromal cells of endometriotic implants, being more expressed in the epithelium [18, 42]. Moreover, endometriotic cells can synthesize and secrete VEGF [42].

Activated peritoneal macrophages and neutrophils also have the capacity to produce and secrete VEGF [18, 43, 44]. Some studies demonstrated that the expression and concentration of VEGF are increased in tissue from endometriotic patients [45–49]. Endometriomas and red implants show the highest concentrations of VEGF [45, 46]. The expression and secretion of VEGF from human endometrial stromal cells are modulated by activin A [30].

### 3.2. Peritoneal Fluid from Patients with Endometriosis

The peritoneal fluid of patients with endometriosis is a complex suspension carrying inflammatory cytokines, growth factors, steroid hormones, proangiogenic factors, macrophages, and endometrial and red blood cells [42, 43, 50–52]. Leukocytes circulating in the peritoneal fluid of patients can produce and release high amounts of VEGF [18, 43, 44]. Moreover, the peritoneal fluid concentrations of VEGF in patients with endometriosis correlate with the stage of the disease [42]. Other proangiogenic factors, namely, IL-8 [30, 53–56], hepatocyte growth factor (HGF) [57, 58], erythropoietin [59], angiogenin [60], macrophage migration inhibitory factor [61], neutrophil-activating factor [62], and TNF-*α* [63, 64], are all found at increased concentrations in the peritoneal fluid of patients with endometriosis. This proangiogenic milieu is reinforced by reduced concentrations of antiangiogenic factors, such as adiponectin [65] and interferon-gamma-induced protein 10 (IP-10) [66, 67], although levels of the endogenous VEGF antagonist soluble Flt-1 were reported to be increased in the pelvic fluid of endometriosis cases [68].

### 3.3. Agents with Antiangiogenic Properties

As one of the most potent angiogenic factors, VEGF is postulated to be involved in the progress of the ectopic lesions in endometriosis [22, 67]. Vascularization and VEGF and its receptor expression are particularly high in deeply infiltrating endometriosis, supporting the hypothesis that antiangiogenic therapy (Table 1) could represent a new and promising modality of treatment of this symptomatic disease manifestation [13]. Classic treatments of endometriosis rely on the use of hormonal drugs with undesirable menopausal side effects or surgery, with its risks of complications, frequent recurrence, and common need for adjuvant medical therapy. New agents, like antiangiogenic factors, offer a different perspective in endometriosis therapy, but their development will necessitate the monitoring of potential side effects.

### 3.4. VEGF Blockers and Inhibitors

Soluble truncated VEGF receptors (Flt-1) and affinity-purified goat antibodies to human VEGF-A inhibited the growth of human endometrium fragments implanted into nude mice [69]. In similar studies, treatment with anti-human VEGF antibody resulted in a significant decrease in the number of lesions of endometriosis in the nude mouse model [70]. The angiogenesis inhibitors TNP-470, endostatin, and anginex inhibited the number of endometriosis lesions present in a mice model [70]. Lodamin, an oral nontoxic formulation of TNP-470, suppressed the mobilization of circulating endothelial cells and endothelial progenitor cells and inhibited the growth of endometriotic lesion in a mouse model of endometriosis, demonstrating a potential clinical use of antiangiogenic therapy for endometriosis [19]. 

Bevacizumab, a full-length recombinant humanized monoclonal antibody that inhibits VEGF, inhibited the development and cell proliferation in endometriotic lesions, reduced vascular density, increased apoptosis, and reduced VEGF levels in peritoneal fluid in a murine model of endometriosis [71]. Bevacizumab reduced the volume of endometriotic implants but did not show any detrimental effect on ovarian reserve in a rat model of induced endometriosis [72].

Sorafenib, another anti-angiogenic agent, is an orally active multikinase inhibitor that interferes with the activity of the VEGF receptor, along with other tyrosine kinase receptors. This drug reduced the microvessel density and lesion volume of endometrial implants in a rat model of induced endometriosis [72]. 

Hypoacetylation of histone H4 is associated with downregulation of the p53 and von Hippel-Lindau proteins and the upregulation of HIF-1*α*. All three effects promote VEGF gene expression [73]. Romidepsin, a histone deacetylase inhibitor, may be a potential therapeutic candidate against angiogenesis in endometriosis. This agent inhibited VEGF gene transcription, protein expression, and secretion of VEGF in an *in vitro* study with human immortalized epithelial endometriotic cells [74]. 

Lipoxin A4 (LXA4) is an endogenous eicosanoid which participates in the regulation of inflammation. This lipid can block migration of endothelial cells and VEGF-stimulated angiogenesis [75]. In endometriosis induced in BALB/c mice, LXA4 reduced the endometriosis lesion size and downregulated inflammation-associated proteins, including IL-6 VEGF and matrix metalloproteinase 9 [76]. 4-Hydroxybenzyl alcohol (HBA) is a naturally occurring phenolic compound, found in many plants, including carrots [77]. HBA exhibits an anti-inflammatory activity and the development of new blood vessels [78]. HBA inhibited the initiation of the angiogenic process by downregulating VEGF and matrix-metalloproteinase-(MMP-) 9 expression and by affecting endothelial cell migration *in vitro* and *in vivo* [78, 79]. 

Parecoxib, a selective cyclooxygenase-2 (COX-2) inhibitor, reduced lesion size, microvessel density, the number of macrophages, and the expression of VEGF and led to atrophy and regression of endometrial implants in a rat model of peritoneal endometriosis [80].

The major constituent of green tea, *Epigallocatechin gallate*, also appears to have antiangiogenic properties since its use decreased endometriotic lesion size, microvessel diameter and density, and VEGF mRNA expression in an experimental SCID mouse model of endometriosis [81]. Moreover, this extract from green tea increased apoptosis in the endometriotic lesions [81]. Another study confirmed that *Epigallocatechin gallate* blocked VEGF expression of hamster endometrial cells *in vitro* and inhibited angiogenesis and blood perfusion of endometriotic lesions *in vivo*, inducing regression of the endometriotic lesions [82]. These antiangiogenic and proapoptotic proprieties of green tea suggest that it might be used as a complementary treatment in endometriosis, but its potential benefit remains to be evaluated in clinical trials. Combined inhibition of VEGF, fibroblast growth factor, and platelet-derived growth factor by inhibitor SU6668 suppresses angiogenesis and vessel maturation in endometriotic lesions in an animal model [22]. 

Macrophage migration inhibitory factor (MIF), which is markedly upregulated in active endometriosis lesions [83], also contributes to angiogenesis. An MIF antagonist suppressed the development of endometriotic lesions *in vivo* reducing the expression of VEGF, cell adhesion receptors, MMP-2, MMP-9, IL-8, and cyclooxygenase- (COX-) 2. Moreover, MIF antagonist demonstrated a proapoptotic action in the nude mouse model [84].

### 3.5. Other Antiangiogenic Agents

Retinoic acid, known to have anti-angiogenic proprieties, decreased the volume of endometriotic implants in mouse [85] and rat [72] models of induced endometriosis. Xanthohumol, a prenylated flavonoid isolated from hops, demonstrated the capacity to inhibit the formation of new blood vessels in developing peritoneal and mesenteric endometriotic lesions which were surgically induced in BALB/c mice, without affecting the histomorphology of the uterus or ovary [86]. Rapamycin, an immunosuppressant drug with antiangiogenic effects, induced regression of endometriotic lesions by inhibiting neovascularization and cell proliferation in an *in vitro* model [87].

Progestogens (progesterone, dydrogesterone, or its metabolite dihydrodydrogesterone) reduced proliferation of endometrial stromal cells and suppressed the transcription of VEGF-A and the microvessel density in human ectopic endometrial lesions in a mouse model, regulating important factors for the establishment of ectopic lesions [88]. Dienogest reduced IL-1*β* production from peritoneal macrophages and implant volume in a rat model of endometriosis [89].

Statins are inhibitors of 3-hydroxy-3-methylglutaryl-coenzyme A (HMG-CoA) reductase with intrinsic antioxidant, anti-inflammatory, and anti-angiogenic properties [90]. Atorvastatin inhibited the inflammatory and angiogenic genes COX-2 and VEGF in endometriotic stromal cells [91]. Cell proliferation and angiogenesis were inhibited by lovastatin in a dose-dependent manner in a three-dimensional *in vitro* model of endometrium [92].

 The dopamine agonist cabergoline exerts antiangiogenic effects through VEGFR-2 inactivation inhibiting the growth of established endometriosis lesions [93]. Moreover, cabergoline treatment results in a significantly lower expression of VEGF and VEGFR-2 in endometriotic lesions [94]. Quinagolide, binding to dopamine D2 receptor, downregulated VEGF/VEGFR2, inhibited neoangiogenesis, and reduced the size of active endometriotic lesions [95].

## 4. Conclusion

**Figure 1:**
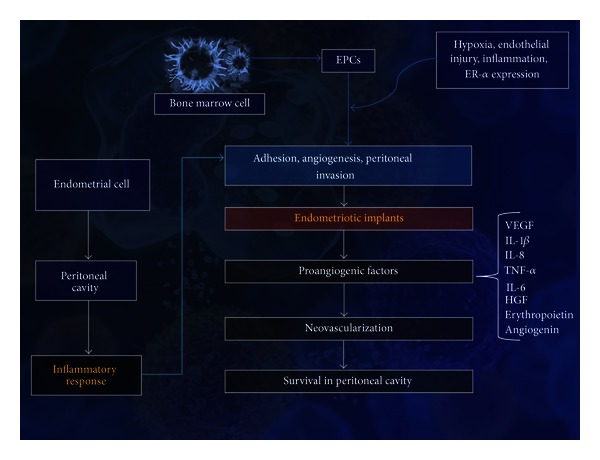
Angiogenesis in the pathogenesis of endometriosis. EPCs, endothelial progenitor cells; VEGF, vascular endothelial growth factor; IL, interleukin; TNF, tumor necrosis factor; HGF, hepatocyte growth factor.

A comprehensive synthesis of the complex pathogenesis of endometriosis remains elusive, but we know that this is a multifactorial disease in which the development and maintenance of endometriotic implants depend on their invasive capacity and angiogenic potential (Figure 1). 

As angiogenesis represents a critical step in the establishment and pathogenesis of endometriosis, this process has been viewed as a potential new target for therapeutic intervention. In this review, experimental studies of several anti-angiogenic agents demonstrated the regression of endometriotic lesions by reducing their blood supply (Table 1). Further studies are necessary before these novel agents can be introduced into clinical practice, in particular the establishment of the safety of anti-angiogenic medications in women who are seeking to become pregnant. Precautions such as those instituted for the prescription of retinoic acid should be considered to avoid the possible consequences of impaired blood vessel formation to the developing embryo and placenta. With this provision, anti-angiogenic treatments offer novel perspectives and mechanisms and promise more effective adjuvant therapies for patients with endometriosis.

## Figures and Tables

**Table 1:** Antiangiogenic agents.

	Antiangiogenic agents	Functional activity (*in vivo* and *in vitro* studies)
*VEGF blockers and inhibitors *	Soluble truncated VEGF receptors (Flt-1)	Inhibited the growth of human endometrium in mice
	Anti-human VEGF antibody	Inhibited the growth of human endometrium and decreased the number of endometriotic lesions
	TNP-470 (lodamin)	Inhibited the number of endometriosis lesions, suppressed the mobilization of circulating endothelial cells and endothelial progenitor cells
	Endostatin and anginex	Inhibited the number of endometriosis lesions
	Bevacizumab (recombinant humanized monoclonal antibody that inhibits VEGF)	Inhibited the development and cell proliferation in endometriotic lesions, reduced vascular density, increased apoptosis, and reduced VEGF levels
	Sorafenib (an orally active multikinase inhibitor)	Interfered with the activity of the VEGF receptor reducing the microvessel density and lesion volume of endometrial implants
	Romidepsin (a histone deacetylase inhibitor)	Inhibited VEGF gene transcription, protein expression and secretion of VEGF
	Lipoxin A4 (LXA4, an endogenous eicosanoid)	Reduced the endometriosis lesion size and downregulated inflammation-associated proteins, including IL-6 VEGF and matrix metalloproteinase 9
	4-Hydroxybenzyl alcohol (HBA, a naturally occurring phenolic compound)	Inhibited the initiation of the angiogenic process by downregulating VEGF and matrix-metalloproteinase-(MMP-) 9 expression and by affecting endothelial cell migration
	Parecoxib (selective COX-2 inhibitor)	Reduced lesion size, microvessel density, the number of macrophages, and the expression of VEGF
	*Epigallocatechin gallate *(major constituent of green tea)	Decreased endometriotic lesion size, microvessel diameter and density, and VEGF mRNA expression
	SU6668	Suppressed angiogenesis and vessel maturation in endometriotic lesions.
	Macrophage migration inhibitory factor (MIF) antagonist	Reduced the expression of VEGF, cell adhesion receptors, MMP-2, MMP-9, IL-8, cyclooxygenase (COX)2

*Other anti-angiogenic agents *	Xanthohumol (a prenylated flavonoid)	Inhibited the formation of new blood vessels
	Rapamycin (an immunosuppressant drug)	Inhibited neovascularization and cell proliferation
	Retinoic acid	Decreased the volume of endometriotic implants
	Progestogens (progesterone, dydrogesterone, or its metabolite dihydrodydrogesterone)	Reduced proliferation of endometrial stromal cells and suppressed the transcription of VEGF-A and the microvessel density
	Statins (atorvastatin, lovastatin)	Inhibited the inflammatory and angiogenic genes COX-2 and VEGF in endometriotic stromal cells
	Dopamine agonists	Reduced microvessel density and angiogenic gene expression
